# Assessing and mining grain amaranth diversity for sustainable cropping systems

**DOI:** 10.1007/s00122-025-04940-w

**Published:** 2025-07-03

**Authors:** Markus G. Stetter, Dinesh C. Joshi, Akanksha Singh

**Affiliations:** 1 Institute for Plant Sciences, University of Cologne, Cologne, Germany; 2 ICAR-Vivekananda Institute of Hill Agriculture (Vivekananda Parvatiya Krishi Anusandhan Sansthan), Almora, Uttarakhand India; 3 Cluster of Excellence on Plant Sciences, University of Cologne, Cologne, Germany

## Abstract

Global challenges and new demands require adaptation of cropping systems. Plant genetic diversity can contribute to adapt and improve crops and create more sustainable agricultural systems. In order to harness this diversity, a unified framework that combines genomic, ecological, and geographical approaches is needed for targeted conservation efforts and breeding strategies. In this review, we discuss the potential of genetic diversity to improve the nutritious and resilient pseudocereal grain amaranth. We emphasize on the utilization of within crop diversity and crop wild relatives. We discuss the impact of hybridization and introgression in facilitating the exchange of genetic material between wild and cultivated amaranth, highlighting their significance in broadening the crop’s genetic base. Additionally, we focus on utilization of climate distribution models in predicting the future geographic ranges and their suitability with implications for conservation and future sustainability. We aim to suggest a roadmap for leveraging genetic diversity of underutilized crops to contribute to resilient and sustainable cropping systems in a changing climate.

## Introduction

With the advances in agronomic technologies and improved varieties, the overall crop production has strongly increased in the last century. However, the predicted increase in human population along with progressing urbanization and climate change, would lead to a global increase in food demand of 35–62% by 2050 (Van Dijk et al. 2021). In the past, the green revolution has enabled a strong overall production increase. Yet, this has favored a small number of crops and led to a decrease in crop variation and increased dependency of global food systems on a limited number of cosmopolitan crop species. This limitation of crop species makes our cropping systems more vulnerable and has implications for food security (Zuza et al. 2024). In addition to the need of enhanced total production, increased nutritional quality of food and ecological services of cropping systems are required (Zuza et al. 2024).

While most of the caloric requirements of humans are fulfilled by a few major crops (i.e., wheat, rice and maize), they often lack essential (micro) nutrients and amino acids (Pedersen et al. 1990). A number of traditional crops with high nutritional value are known, and might have a high potential to improve nutritional quality and ecological requirements of food production systems. Many such crops, often underutilized and neglected in mainstream agriculture, could play a crucial role in nutritional security by providing multiple micro-nutrients, vitamins, and proteins that are often lacking in staple crops (Pedersen et al. 1990; Tadele 2019). Their adaptability to marginal environments and resilience to climatic stresses make them vital for sustaining health and food systems, particularly in vulnerable regions (Tadele 2019; Pulvento and M. h. Sellami, and A. Lavini, 2022). Yet, these crops have received little attention and improvement in the last decades (Tadele 2019).

Grain amaranth *(Amaranthus spp.)*, a C$_4$ dicot pseudocereal, is one such minor crop recognized for its high nutritional value, high protein content, and tolerance to harsh environments (Das 2016; Joshi et al. 2018). It can be grown on marginal land and in diverse climates, from tropical lowlands to temperate highlands, with an optimal growth temperature of 25–33 $^\circ$C (Joshi et al. 2018; Santra et al. 2024). The plants are herbaceous, growing up to 300 cm in height. They flower within 4–8 weeks after germination, and seeds start to mature within approximately 35–45 days after flowering (Joshi et al. 2018). The seeds are small, approximately 1 mm (0.9–1.7 mm) in diameter and 0.6–1.3 mg in weight (Mukuwapasi et al. 2024). The seed color varies, ranging from creamy white to brown, gold, and pink, with total yield, ranging from 1500 to 7200 kgs per hectare, depending on variety (Mukuwapasi et al. 2024; Santra et al. 2024).

Amaranth contains a balanced amino acid and micro-nutrient profile with bio-active flavonoids and betalains. Shukla et al. (2018) analyzed nutritional quality in 1309 amaranth accessions and found that total protein content was greater than 10%, with high levels of essential amino acids like lysine and methionine, which are often limited in conventional cereals. Its gluten-free nature makes it an important dietary component for people with celiac disease or gluten-intolerance, addressing nutritional deficiencies often associated with such dietary restrictions (Joshi et al. 2018; Yadav and Yadav 2024). Besides, grain amaranth is also a rich source of micro-nutrients such as iron, zinc, magnesium, manganese, potassium, and calcium (Joshi et al. 2018). The inclusion of amaranth in food security programs has demonstrated positive health outcomes, with enhanced micro-nutrient intake and child nutrition (Tibagonzeka 2014; Macharia-Mutie et al. 2013; Yadav and Yadav 2024). Furthermore, its adaptability to drought-prone and nutrient-poor soils (de la Rosa et al. 2009; Das 2016; Pulvento and M. h. Sellami, and A. Lavini, 2022; Yadav and Yadav 2024) makes it an ideal candidate for cultivation in areas facing environmental and resource constraints. Recent research advances aim to integrate amaranth into modern cropping systems, enhancing its ecological and economic viability.

In this review, we summarize the current knowledge on the role of genetic diversity and wild relatives of grain amaranth and provide suggestions on how these resources can be integrated to improve the sustainability of food production systems. We discuss the role of introgression and incompatibility in shaping grain amaranth diversity. We explore the potential of genetic diversity from regions beyond a crop’s center of origin that can serve as natural reservoir of variation and adaptive alleles. We further highlight the use of climate distribution models as a comprehensive framework for harnessing genetic diversity in breeding programs and conservation strategies to tackle global food security and improve climate resilience. Our perspectives are transferable and applicable to the large number of minor crops that have been declining over the last centuries but bare great potential for the future.

## Grain amaranth gene pools

Grain amaranth belongs to the family Amaranthaceae consisting of about 40–70 species (Sauer 1967; Stetter and Schmid 2017; Waselkov et al. 2018) including crop, weedy and wild species. Several species in the genus are of high economic importance either because of their use as vegetable and grains or as invasive species. The genus *Amaranthus* is taxonomically classified into three subgenera: *Amaranthus Albersia, Amaranthus Acnida*, and *Amaranthus Amaranthus* (Costea and DeMason 2001). The *Amaranthus Amaranthus* subgenus consists of cultivated grain amaranths (*A. caudatus, A. cruentus* and *A. hypochondriacus*) and wild (*A. hybridus, A. quitensis* and *A. powelli*) amaranths (Stetter et al. 2016; Waselkov et al. 2018). Other species like *A. tricolor, A. viridis* and *A. blitum* along with *A. cruentus, A. dubius, A. albus*, and *A. hybridus* are frequently cultivated as a leafy vegetable. Being rich in micro-nutrients and essential amino acids, both the seeds and leaves of these species are recognized as valuable food sources (Rastogi and Shukla 2013). In addition, *A. palmeri* along with *A. tuberculatus, A. rudis*, and *A. retroflexus* are particularly well known for their resistance to multiple herbicides, causing substantial yield losses in North America (Steckel and Sprague 2002; Bensch et al. 2003).

**Fig. 1:**
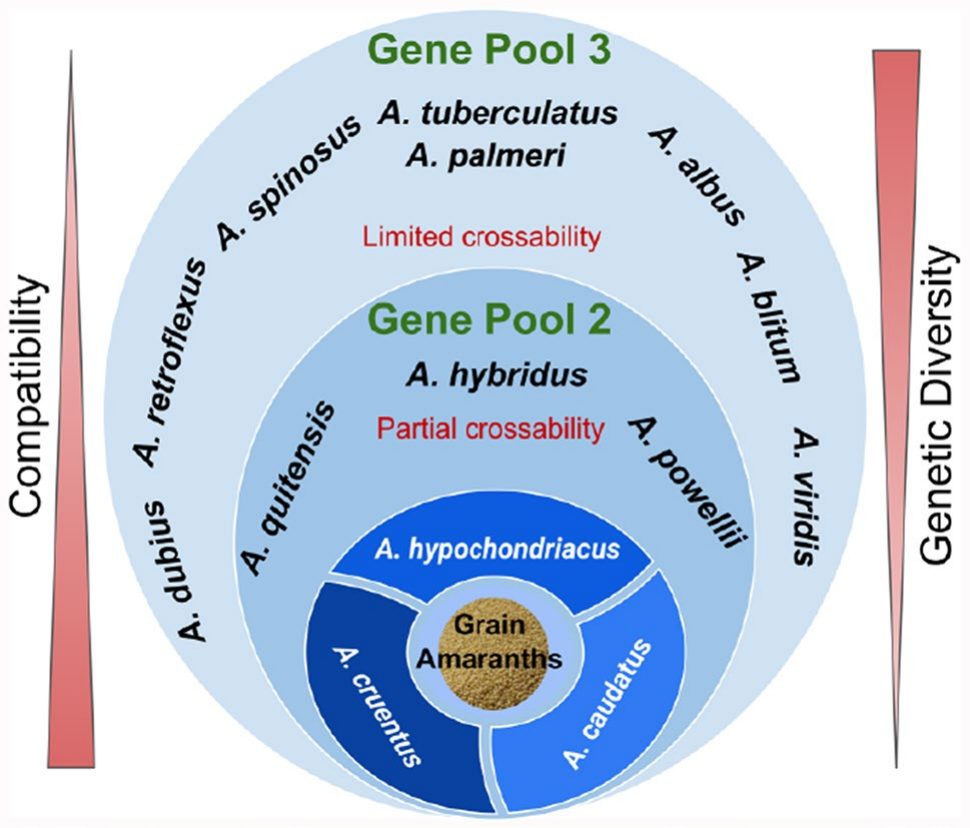
Overview of gene pools for grain amaranth. There are three species of grain amaranth (innermost circle with dark blue). Each of the species along with its local landraces and varieties, is within itself a primary gene pool. Cross-compatibility and partial incompatibility of each of the three grain species also make them a secondary gene pool for the others. Other wild relatives with limited crossability or bridging through secondary gene pools constitute the tertiary gene pools

Within the Amaranthaceae family, species are majorly monoecious with a few being dioecious. The grain amaranths are annual, monoecious and mostly selfing, diploids (2*n* = 32 and 34) with comparatively small genome size of approximately 500 Mbp (Stetter and Schmid 2017). The five species in the ’hybridus complex’ (Stetter and Schmid 2017) can cross with an outcrossing rate of up to 30% (Jain et al. 1982; Hauptli and Jain 1985; Nyambo et al. 2024; Stetter et al. 2016). Some of the weedy wild relatives, including *A. powelli, A. retroflexus, A. palmeri* and *A. tuberculatus* are known to show some compatibility with the grain amaranth species, particularly with their wild ancestor *A. hybridus*, making them suitable secondary and tertiary gene pools (Fig. 1) (Sosnoskie et al. 2009; Nyambo et al. 2024). Brenner et al. (2019) made an attempt to classify the known weedy and wild species from North America into gene pools, including species where compatibility is less clear and partially speculative. In addition, there are approximately 20 other wild weedy species of amaranth growing around the world beyond the native range of the crops with different ecological niches, where the cross-compatibility is not yet known. The high species diversity and adaptability of members of the *Amaranthus* genus create an extensive resource for the crop that should be further investigated to categorize the gene pools (Fig. 1).

## Grain amaranth: wild to domesticated

The archaeological records of grain amaranth date its earliest cultivation to more than 8000 years ago in the two regions of the Americas, Central America and the Andes (Sauer 1967; Brenner et al. 2000). Later, it was cultivated primarily by the Aztecs, Mayans, and Incas as a staple crop alongside maize and beans (Brenner et al. 2000). However with the Spanish conquest, amaranth cultivation declined significantly, potentially due to its use in indigenous rituals (Sauer 1967; Brenner et al. 2000). Interestingly, the domestication of amaranth occurred three times in three different regions of the Americas (Sauer 1967). Two of the species, *A. hypochondriacus* and *A. cruentus*, were domesticated in Central America, while *A. caudatus* was domesticated in South America along the Andes (Sauer 1967). All three species were most likely domesticated from the same common ancestor, *A. hybridus* (Stetter et al. 2020, 2017). While some work suggested *A. quitensis* as the progenitor of the South American grain species *A. caudatus* (Sauer 1967), others suggested it to be an escaped or feral *A. caudatus* (Kietlinski et al. 2014; Stetter et al. 2020). Yet, the status of *A. quitensis* as wild or a feral species remains unclear.

Similar to other crops, the shift from wild populations to cultivated grain amaranth forms has led to a loss of genetic variation (Stetter et al. 2020; Singh and Stetter 2024). This shift from wild to domesticate is a continuum that results in a range of variation in the diversity creating a set of primary gene pools consisting of landraces and modern cultivars. Since, there are three domesticated grain species which can hybridize with each other, each primary gene pool acts as secondary gene pool for the other species (Fig. 1). Although the three grain amaranths can hybridize, they show unequal incompatibility that might have shaped the current crop diversity (Gonçalves-Dias et al. 2023). While Gonçalves-Dias et al. (2023) observed considerable introgression between *A. cruentus* and *A. caudatus*, crosses between accessions of these species suggested strong reproductive barriers. They hypothesized that this barrier is likely due to post-domestication evolution of genetic incompatibilities. No signs of incompatibility were observed between the Central American (*A. hypochondriacus*) and South American (*A. caudatus*) species (Gonçalves-Dias et al. 2023), making them promising heterotic pools for hybrid breeding.

Amaranth exhibits a rather fluid domestication continuum, where cultivated and wild populations continued to exchange genetic material through hybridization and gene flow (Gonçalves-Dias et al. 2023). In many crops, domestication led to the accumulation of mildly deleterious alleles, increasing genetic load (Kim et al. 2021; Rodgers-Melnick et al. 2015; Lu et al. 2006). In grain amaranth, domesticates also showed higher load than their wild relatives, but the introgression from the wild relatives into the crops likely led to a reduction in genetic load in the crop species (Gonçalves-Dias et al. 2023). Such a dynamic genetic exchange with diverse wild relatives might have helped maintain adaptive potential in grain amaranths, but potentially led to the lack of some domestication traits. The high diversity provides an opportunity to harness both cultivated and wild genetic resources to breed resilient and climate-adapted varieties.

## Genetic resources and genomic tools in Amaranth

Amaranth consists of rich *ex-situ* germplasm collections from diverse regions. Currently, there are more than 60 different germplasm conservation centers, spanning more than 11 countries with collected accessions reaching more than 11,000 (Das 2016; Joshi et al. 2018). These provide a great resource for assessing and utilizing their adaptive potential, representing different regions with local variability. Although a large number of gene bank accessions are being characterized phenotypically (Boro et al. 2024; Baturaygil and Schmid 2022; Yeshitila et al. 2024), only a sub-set of these accessions have been characterized genetically (Wu and Blair 2017; Stetter et al. 2020; Singh and Stetter 2024). However, recent advances have greatly increased the number of genotyped individuals. Genotypic germplasm characterization is of particular importance as taxonomic species delimination is complicated by the overlapping morphological traits (Singh and Stetter 2024; Costea et al. 2006; Brenner et al. 2010). However, advances in genetics are improving species delineation, paving the way for more accurate classification and effective utilization of genetic resources in breeding efforts. The development of a set of genetic markers for species delineation would be useful for improved and economical characterization of these genebank accessions.

In recent years, new genome sequencing methods have enabled the characterization of many plant species, including amaranth. The first draft genome assembly for amaranth (*A. hypochondriacus*) was published in 2014 (Sunil et al. 2014). Lightfoot et al. (2017) published a first high-quality chromosome-scale assembly for *A. hypochondriacus* which was recently further improved (Winkler et al. 2024; Graf et al. 2025). Currently, unlike most other minor crops, amaranth has a well-assembled and annotated genome for 2 grain crop species and wild, weedy, and leafy species (Winkler et al. 2024; Montgomery et al. 2020; Wang et al. 2023; Graf et al. 2025), making its genetic characterization and promotion easier for further genetic improvement. Recently, Deb et al. (2020) suggested a cost-effective method for creating genome assemblies for local landraces from low-coverage long-read sequencing and public data. This provided a valuable reference for crop improvement in South Asia that is being utilized for eco-TILLING and TILLING-based approaches to identify beneficial mutations for several agronomic and nutritional traits (Deb et al. 2020). Beyond, Graf et al. (2025) presented the first chromatin and methylation landscape map for grain amaranth, representing the functional space of the genome. They also compared the chromatin accessibility patterns during amaranth domestication showing species-specific changes, indicating an epigenetic layer of diversity that could be utilized for further functional characterizations of agronomically important traits.

The high-quality genomic data and the resources providing access to these data can accelerate the improvement of grain amaranth. To promote the availability and accessibility of population genetic values, including estimates of genetic diversity, a genome browser ’PopAmaranth’ is available (Gonçalves-Dias and Stetter 2021, amaranthgdb.org/popamaranth.html). The browser is based on a curated set of 88 morphologically and genetically diverse accessions (Gonçalves-Dias and Stetter 2021) that could be considered a core collection for grain amaranth. Another genomic genome browser called ’Amaranth genomic resource database’, combining BLAST search, genes, transcription factors, and miRNAs is available (Singh et al. 2023, www.nbpgr.ernet.in:8080/AmaranthGRD/). These multidisciplinary platforms will help build a research community, facilitating interdisciplinary research with focus on amaranth improvement.

The availability of amaranth genome assemblies accelerated whole-genome (re-)sequencing studies that enhanced our understanding of its domestication, expansion and evolutionary history (Stetter et al. 2020; Gonçalves-Dias et al. 2023; Singh and Stetter 2024; Graf et al. 2025). Genome sequences of amaranth accessions not only enabled precise genetic mapping, but facilitated the identification of key genes for important traits. For instance, bulked segregant analysis has revealed the causal mutation in an enzyme for betalain pigmentation (Winkler et al. 2024). This was enabled by the high-quality genome annotation and whole genome re-sequencing. The genetic control of few other traits has also been successfully mapped using genome wide-markers (Stetter et al. 2020; Jamalluddin et al. 2022; Wang et al. 2023). Additional genotyping of diverse panels, along with high-quality phenotypic measurements, will allow the identification of loci that control agronomically important traits. Further comprehensive assessment of amaranth diversity panels in common garden experiments (de Villemereuil et al. 2016), incorporating local germplasm alongside global crop representation, will enable the identification of adaptive traits, the assessment of their heritability, and an analysis of significant marker-trait associations.

In the future, the development of high-quality ’pangenomes’ from a diverse set of core reference accessions would be useful for cross-species genotyping. Pangenomes would also facilitate the identification of structural variants, including large insertions, deletions and copy number variation, beyond single nucleotide polymorphisms (SNPs),. The integration of additional ’omics’ data can increase the efficacy of identifying causal loci for relevant traits.

## Harnessing genetic and phenotypic diversity in grain amaranth for global adaptive breeding

### Genetic diversity

The diversity preserved in the available genetic resources form the foundation for crop improvement. In recent years, a large number of grain amaranth and its wild relatives have been genetically characterized at the whole genome level, revealing a high genetic diversity within and between species (Stetter et al. 2020; Wu and Blair 2017; Singh and Stetter 2024; Hoshikawa et al. 2023; Kreiner et al. 2022). Within the crop’s native range, several alleles were found to be private to each species (Wu and Blair 2017), suggesting for differences in diversity that can be utilized for marker assisted breeding. As expected, whole genome re-sequencing studies have shown an elevated genetic diversity in the wild relative of amaranth with comparatively higher number of private alleles (Wu and Blair 2017; Stetter et al. 2020; Singh and Stetter 2024). But substantial numbers of private alleles were also identified in the global grain crop accessions, predictive of potential local diversity (Stetter et al. 2020; Singh and Stetter 2024). The three grain species and the wild species showed independent signals of selection that point towards enhanced cross-species adaptive potential when combining breeding pools (Stetter et al. 2020; Singh and Stetter 2024). Deeper mining of these specific variations would help broaden the available genetic resources.

**Fig. 2:**
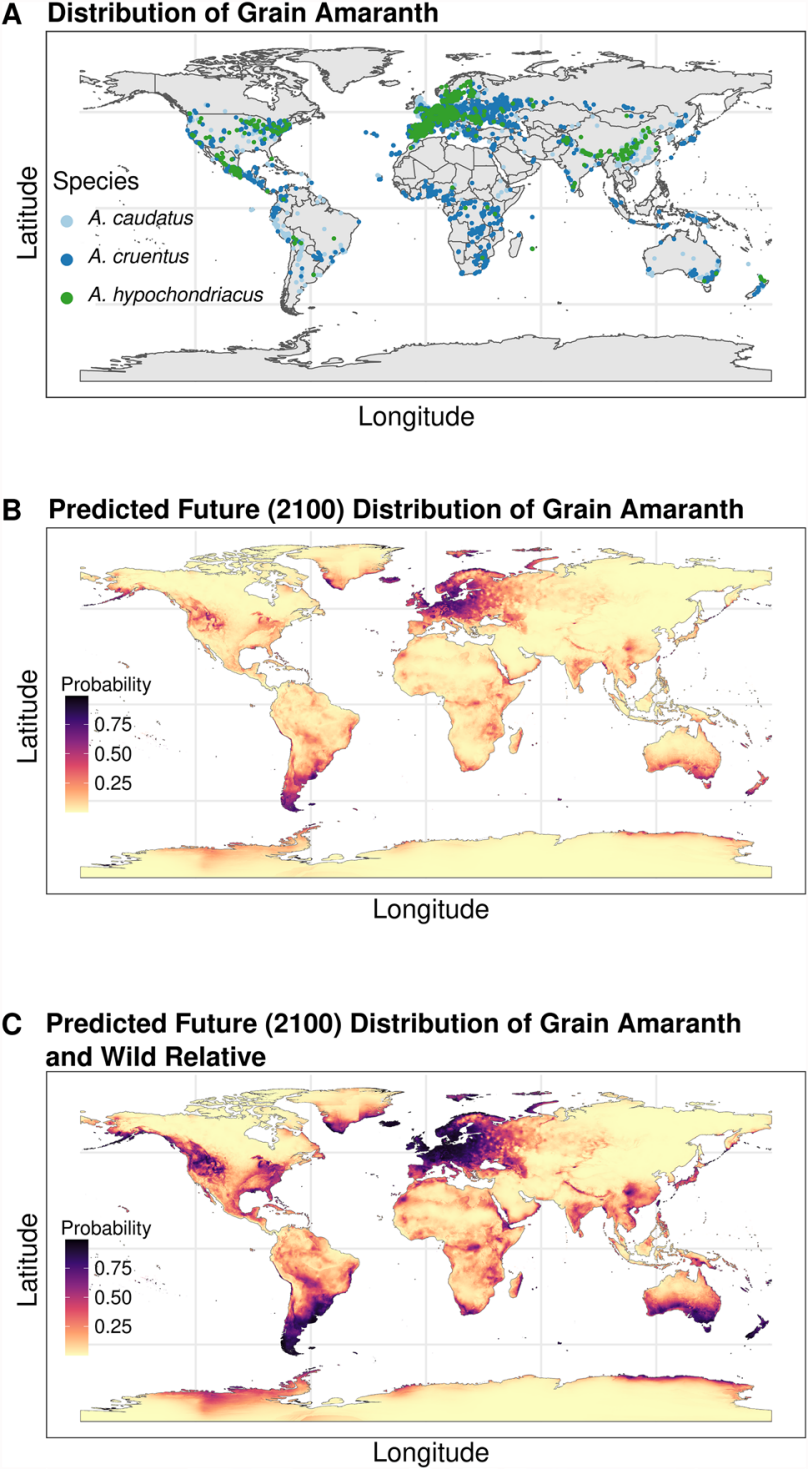
Usability of climatic distribution models. **A** Geographic distribution of three grain amaranth species. **B** Probability of occurrence of three grain amaranth species based on current occurrence of the three species and predicted future climate for year 2100. **C** Probability of occurrence of grain amaranth species based on current occurrence of the three species and its three wild relative (*A. hybridus, A. quitensis* and *A. powellii*) and predicted for future climate (2100). Climate data was downloaded from Worldclim database (https://www.worldclim.org/) at resolution of 2.5 min. The data for occurrence were extracted from the GBIF occurrence database (doi: https://doi.org/10.15468/dl.ke35yd and https://doi.org/10.15468/dl.e9f63f; GBIF is based on observation data, which likely leads to an over-representation of regions with higher study input, as suggested by the high density of grain amaranth in Europe, while the crop is mostly grown in other regions)

Beyond its domestication center, grain amaranths show a remarkable occurrence throughout the globe across diverse climates and environments (Fig. 2A). Originating in the Americas, where it was cultivated as a staple crop by ancient civilizations, Amaranth has since spread to Africa, Asia, and beyond (Das 2016). Grain amaranth has successfully established in these new introduced regions, with little loss of genetic diversity (Singh and Stetter 2024). Unlike other crops, where introduction to similar locations induced increased admixture (Yang et al. 2012; Bellucci et al. 2023), grain amaranth has maintained its genetic identity with no identifiable admixture (Singh and Stetter 2024), ensuring genetic integrity and adaptability for breeding programs. This global presence has resulted in the development of a wide range of varieties and landraces adapted to local environmental conditions and cultural practices. For instance in India, the analysis of large-scale whole-genome sequencing data from grain amaranth has shown the maintenance of genetic diversity, despite an introduction bottleneck(Singh and Stetter 2024). Moreover, the chromosome-level assembly generated from a local landrace of *A. hypochondriacus* in India harbors considerable differences from a cultivar from the Americas, but similarity with nearby local landraces (Deb et al. 2020). Hence, the different locally adapted landraces across the globe likely carry alleles beyond those present in the centers of domestication.

### Phenotypic diversity

In addition to the high genetic diversity, amaranth exhibits high morphological and agronomic diversity potentially due to its wide global distribution. Phenotypic evaluations across introduced regions highlight the adaptability of amaranth to diverse environments. Assessment of phenotypic traits from a wide global collection showed an association of traits with the local environmental conditions, based on which genotypes were selected as candidates for local breeding programs (Wu et al. 2000; Boro et al. 2024; Baturaygil and Schmid 2022). Boro et al. (2024) phenotyped 900 accessions and identified 251 core accessions for further phenotypic characterization. They measured 17 phenotypic traits in two different common gardens in two years and identified high variability in traits with high heritability in the two tested locations. Based on the phenotypic analysis, they identified 23 accessions showing consistent high yield. These accessions would be suitable to be grown in the tested conditions. By further summarizing phenotypic variability, 19 elite accessions with early flowering, high seed yield, and elevated seed protein content were selected to be suitable for breeding superior cultivars (Boro et al. 2024). Significant variability in important agronomic traits has also been observed among Ethiopian amaranth genotypes (Yeshitila et al. 2024), providing a valuable resource for future regional breeding efforts. The evaluation of flowering time in 253 genebank accessions have revealed the adaptation of amaranth to different photoperiods, which is crucial to optimize the cultivation across diverse environments (Baturaygil and Schmid 2022). Moreover, Pulvento and M. h. Sellami, and A. Lavini, (2022) studied the effect of drought and salinity on seed quality and yield and suggested that grain amaranth can be grown in marginal European environments where traditional crops cannot grow. These findings highlight the vast diversity of amaranth, underlining its potential for regional adaptation and resilience in diverse environments, making it a promising candidate for future breeding programs.

Crop wild relatives of amaranth can serve as valuable sources of agronomically important traits that can be utilized to enhance crop performance through trait introgression. For example, Wu et al. (2000) evaluated 229 genotypes from 20 *Amaranthus spp.* under two different common garden conditions and showed that the wild relatives of amaranth represent a greater variability in phenotypes, with reduced photoperiod sensitivity and greater resistance to diseases. Such variation can be exploited to reduce the photoperiod sensitivity, particularly for *A. caudatus*, which showed delayed flowering and maturity under temperate conditions (Wu et al. 2000). An example of the use of wild relatives for trait transfer in amaranth is the successful incorporation of the non-dehiscence from *A. powellii* into *A. hypochondriacus* and *A. cruentus*, reducing seed shattering (Brenner 2002). The observed herbicide resistance in *A. hybridus* (Trucco et al. 2006) and other weedy- wild relatives (Yanniccari et al. 2022) might be transferred into the crops species to facilitate large scale production. Further, an in-depth characterization of the available diversity in wild relatives will enhance their utilization in future breeding programs.

While the availability of genome sequences is rapidly growing, increased high-quality phenotyping in a range of environments will be essential to harness the phenotypic and genetic diversity of grain amaranth. The integration of genomic data and multi-location phenotypic data into breeding strategies can enable the rapid improvement of the nutritious crop for future global and local agricultural systems.

## Enhancing grain amaranth resilience through climate distribution models

Traditional assessment approaches, like empirical multi-location field trials are powerful to identify the response of individual genotypes to climate variables. However, they are limited in their capacity, are time-consuming, resource-intensive, and possess little ability to predict adaptation to future climate scenarios. Climatic distribution models have emerged recently as a powerful tool to identify climatic factors, influencing species distribution and predicting suitable habitats for different species, including crops under current and future climate scenarios (Elith and Leathwick 2009; Estes et al. 2013; Ali et al. 2024). Escobedo-López et al. (2014) tested the future climatic suitability of cultivation for the three grain amaranths and their wild relatives. They identified temperature as a major factor governing the differential distribution of the species and predicted a decrease in suitable areas for cultivation of grain amaranth in future. Similarly, Pulvento et al. (2015) assessed the adaptability of amaranth in Mediterranean environments under different climate scenarios, predicting temperature-dependent alterations of the growth cycle as a critical determinant of grain yield. The integration of empirical productivity estimates correlated well with the climatic suitability of the species (Estes et al. 2013; Pulvento et al. 2015), suggesting the usefulness of such models and their further adjustments to improve predictions.

Species distribution models have identified significant differences in suitable cultivable areas for grain species and wild amaranth, where the predicted future suitable area for grain amaranth cultivation was reduced compared to the current area (Escobedo-López et al. 2014). The incorporation of genetic resources form wild relatives into breeding programs can enhance environmental adaptation and increase the area for amaranth cultivation in the future (Figure 2B,C). The models have even identified a few areas where the crop has not been grown before to be suitable for future cultivation due to climate change (Arenas-Castro et al. 2020), demonstrating the ability of these methods to predict crop sustainability.

Combining climatic models and multi-location transplant trials revealed that although certain areas may show climatic suitability, actual survival and growth also depend on physiological and genetic constraints (Anderson et al. 2025). With the availability of genetic data climatic models could indentify locally adaptive alleles, offering a predictive framework for future crop adaptation and assisted migration (Singh and Stetter 2024). For instance, Singh and Stetter (2024) showed that certain accessions from India could serve as potential pre-adapted genotypes in predicted climatic conditions in the crops’ domestication centers. A similar assessment of the genetic offset, based on the adaptive landscape in amaranth populations revealed vulnerabilities (Singh and Stetter 2024), and could guide the strategic planning for breeding programs. Conservation efforts can be enhanced by the selection of the target locations, partially based on matching climates at the location of origin and the target area for production (Zhao et al. 2023). Such proactive approaches, coupled with multi-location trails, genomic and ecological data can help safeguarding genetic resources with adaptive capabilities for the future (Anderson et al. 2025).

## Breeding Amaranth through mining genetic diversity

A considerable portion of the diversity evaluated in the cultivated gene pool and wild relatives of amaranth remain underutilized in breeding operations. There are only a limited number of instances of using genetic resources in amaranth breeding programs. The amaranth working collection at the Rodale Research Centre (RRC) in Pennsylvania, USA has been an essential source of economic traits, including lodging resistance, shattering resistance, early maturity, and high grain yield (Stallknecht and Schulz-Schaeffer 1993; Trucco and Tranel 2011). The germplasm lines of the RRC collection constitute the parentage of the majority of contemporary commercial amaranth cultivars released in the USA (Stallknecht and Schulz-Schaeffer 1993). Similarly, in India, commercial cultivars such as Annapurna, PRA-1, PRA-2, and Durga were developed by direct selection from native germplasm lines adapted to the Himalayan region (Raiger and Jajoriya 2023). Nevertheless, only little of the extensive worldwide genetic diversity of grain amaranth has been used in the breeding programs. However, identification of QTL and direct use of genetic diversity could accelerate the breeding of amaranth, allowing to target specific traits with greater precision and efficiency. For instance, recently, a single dominant gene, *Acsh*, has been identified as a key regulator of seed-shattering in *A. cruentus* (Kondo et al. 2024). The use of this gene could accelerate the development of non-shattering grain amaranth cultivars. This could render the crop more suitable for large-scale cultivation and improve its overall harvestability.

Heterosis, the over-performance of offspring compared to their parents has been successfully used in a variety of crops to improve yield and yield stability (Virmani et al. 1982; Duvick 2001). In amaranth, a mid-parent heterosis of up to 88% has been reported in some traits, including grain yield and biomass (Pandey 1984; Lehmann et al. 1991). The cross compatibility observed between the amaranth species (Jain et al. 1982; Hauptli and Jain 1985) could be potentially exploited for hybrid breeding and incorporation of specific traits. However, hybrid breeding is not well established in amaranth. Efforts have been made for interspecific hybridization in amaranth (Brenner et al. 2000; Brenner 2002) to transfer traits from wild *A. pumilus* (increased seed size), *A. powellii* (reduced seed shattering) and *A. hybridus* (herbicide tolerance) to grain amaranth. Although hybridization and introgression is very common within grain amaranths (Stetter et al. 2020; Gonçalves-Dias et al. 2023), partial and full incompatibilities observed within and between crop species (Pal and Khoshoo 1972; Kole 2011; Gonçalves-Dias et al. 2023), warrants for a systematic assessment to identify compatible valuable lines as input for breeding programs. Consequently, pre-breeding initiatives augmented by advanced regeneration procedures (Locy and Fisher 1985; Castellanos-Arévalo et al. 2020) and genome editing (Vollmer et al. 2025), will enable the deployment of essential genes from distant wild species into the cultivated gene pool of amaranth.

Male sterility systems that enable the production of hybrid seeds would further advance genetic gain and commercial viability. Both nuclear and cytoplasmic male sterility with putative restorer genes have been discovered in amaranth (Peters and Jain 1987; Gudu and Gupta 1988). The first cytoplasmic male sterile line for commercial use has been made available but lacks agronomic performance for commercial use (Brenner 2019). Identification of genetic loci and development of specific molecular markers would aid in incorporating male sterility in breeding lines for increased performance.

Advanced Backcross-QTL (AB-QTL) analysis for managing backcross generations and monitoring favorable and unfavorable alleles during introgression has become an effective breeding strategy for a variety of crops (Tanksley and Nelson 1996; Sharma et al. 2013). The recent progress in amaranth genomics, allows the incorporation of pre-breeding by interspecific hybridization to enhance the genetic base of commercial amaranth breeding programs. Stetter et al. (2016) suggested speed breeding and developed efficient crossing methods to reduce breeding cycles of amaranth. Together, these techniques can help introduce AB-QTL introgression and hybrid seed development.

Next-generation phenotyping platforms, augmented by remote sensing and artificial intelligence techniques, will enable the quick and accurate characterization of extensive breeding populations (Li et al. 2021). For instance, automated seed phenotyping systems using optical seed assays (Jahnke et al. 2016; Tanabata et al. 2012) may significantly enhance the screening of large panels for seed size variation in small-seeded crops such as amaranth. Genomic selection, using genomic estimated breeding values (GEBVs) has evolved as an effective method to accelerate genetic improvement for complex traits (Meuwissen et al. 2001). In amaranth, Genomic selection can be used as an effective tool to estimate GEBVs for expensive quality (nutritional) traits and yield related traits. The identification of individual trait loci and the use of genome editing on these together with genomic selection for complex traits has high potential to advance grain amaranth breeding.

## Conclusions and outlook

Grain amaranth has recently gained recognition for its high nutritional value, health benefits, and resilience to harsh growing conditions. The composition of breeding pools underline the high genetic, morphological, and agronomic diversity of grain amaranth. Harnessing the amaranth diversity can enable its use for sustainable agriculture in the face of climate change. Integration of multi-omics into the identification and characterization of germplasm collections will improve the understanding of the genetic control of various important agronomic and quality traits. Performance gaps to major crops can be reduced by integrating genetic diversity and modern breeding techniques through targeted precision breeding into grain amaranth improvement programs (Vollmer et al. 2025). The incorporation of climatic modeling and focused identification of suitable genotypes for target environments could further help in selecting parental lines for local breeding programs. The last decade of amaranth research has strongly improved the resources for grain amaranth and provides a strong foundation for public and private breeding efforts. Through the integration into cropping systems and value chains, amaranth can contribute to an improved food security and sustainability.
